# Review: The use of functional magnetic resonance imaging (fMRI) in clinical trials and experimental research studies for depression

**DOI:** 10.3389/fnimg.2023.1110258

**Published:** 2023-06-27

**Authors:** Vasileia Kotoula, Jennifer W. Evans, Claire E. Punturieri, Carlos A. Zarate

**Affiliations:** Experimental Therapeutics and Pathophysiology Branch, National Institute of Mental Health, National Institutes of Health, Bethesda, MD, United States

**Keywords:** functional magnetic resonance imaging (fMRI), depression, clinical trial, selective serotonin reuptake inhibitor (SSRI), ketamine, amygdala

## Abstract

Functional magnetic resonance imaging (fMRI) is a non-invasive technique that can be used to examine neural responses with and without the use of a functional task. Indeed, fMRI has been used in clinical trials and pharmacological research studies. In mental health, it has been used to identify brain areas linked to specific symptoms but also has the potential to help identify possible treatment targets. Despite fMRI's many advantages, such findings are rarely the primary outcome measure in clinical trials or research studies. This article reviews fMRI studies in depression that sought to assess the efficacy and mechanism of action of compounds with antidepressant effects. Our search results focused on selective serotonin reuptake inhibitors (SSRIs), the most commonly prescribed treatments for depression and ketamine, a fast-acting antidepressant treatment. Normalization of amygdala hyperactivity in response to negative emotional stimuli was found to underlie successful treatment response to SSRIs as well as ketamine, indicating a potential common pathway for both conventional and fast-acting antidepressants. Ketamine's rapid antidepressant effects make it a particularly useful compound for studying depression with fMRI; its effects on brain activity and connectivity trended toward normalizing the increases and decreases in brain activity and connectivity associated with depression. These findings highlight the considerable promise of fMRI as a tool for identifying treatment targets in depression. However, additional studies with improved methodology and study design are needed before fMRI findings can be translated into meaningful clinical trial outcomes.

## 1. Introduction

Functional magnetic resonance imaging (fMRI) is a commonly-used, non-invasive technique used to study the living human brain. fMRI refers to the acquisition of data modulated with extrinsic (e.g., task stimulus) stimuli or allowed to follow intrinsic fluctuations (e.g., thought). It is typically conducted using blood oxygen level dependent (BOLD) sensitivity methods—such as resting-state and task-based MRI.

In pharmacological research, fMRI is mainly used to help develop and validate markers associated with brain dysfunction in specific disorders (Sadraee et al., 2021). Examining these fMRI markers as potential treatment targets can improve our understanding of the impact that certain compounds have on brain function and structure (Carmichael et al., 2018). In clinical trials, fMRI can be used in the earlier stages of drug development to help detect whether the compound under investigation causes central nervous system (CNS) changes in brain regions relevant to the study population. In that respect, fMRI could be a useful tool for the NIMH-proposed “fast-fail” approach where pharmacological compounds are assessed based on their efficacy to modulate the function of specific treatment targets. This approach aims to speed up drug development by quickly discarding compounds that fail to demonstrate efficacy in affecting biomarkers relevant to the disorders they are investigated for (*From Discovery to Cure*, 2010). In later-stage clinical trials, fMRI can help detect the normalization of a disease-related signal at a treatment-relevant dose (Carmichael et al., 2018; Sadraee et al., 2021).

fMRI is the primary tool used in research studies of both patients and healthy volunteers that seek to examine brain function and evaluate drug action in mental health. A great example of the efficacy of fMRI as a primary outcome measure in clinical trials that aims to quickly and reliably investigate pharmacological compounds in mental health disorders is the study by Krystal et al. In this study, fMRI signal changes in the VS (Ventral Striatum) during reward anticipation, were successfully used as a primary outcome measure to assess the efficacy of a κ-opioid receptor in a population with anhedonia and mood or anxiety disorder (Krystal et al., 2020). Despite its many advantages, fMRI is not commonly included as a primary outcome measure in clinical trials, even in those investigating mental health disorders. However, in depression, for example, clinical trials still rely primarily on scales and questionnaires that measure symptom changes before and after the administration of specific compounds with antidepressant action to assess drug efficacy. Such instruments, although important in disease diagnosis and in the identification of symptom changes before and after treatment, offer no insight as to the mechanisms of action of the antidepressant compounds under investigation or the brain areas targeted by these compounds.

In this context, fMRI offers an objective research tool that, if used effectively, could enable not only the characterization of brain-related deficits associated with depression and other mental health disorders but also help identify drug targets and compare brain activity between different drug conditions (Tracey and Wise, 2001; Wise and Tracey, 2006; Carmichael et al., 2018). Such information is critical for determining the specificity of targets that could be investigated as potential biomarkers for these disorders as well as their treatment. Despite these advantages and the technological progress of the field, fMRI research findings still do not easily translate to results with immediate clinical value.

This review seeks to understand the use of fMRI in pharmacological clinical trials and research studies in depression. It focuses on compounds with antidepressant action—mainly selective serotonin reuptake inhibitors (SSRIs) and the glutamatergic modulator ketamine—to identify the brain areas and networks targeted by these compounds that could serve as potential biomarkers of antidepressant response. The results presented here can be used to consider fMRI as a primary outcome measure for clinical trials and experimental research studies. Broader lessons regarding what has been learned from the use of fMRI in clinical trials and research studies are also discussed, and issues that could impede its use as a primary outcome measure in clinical trials are addressed. Finally, future directions that could further enhance the role of the technique in pharmacological studies are proposed.

## 2. Methodology

### 2.1. Study selection

Two clinical trial databases were searched to identify clinical trials of pharmacological compounds that target depression and have used fMRI: clinicaltrials.gov (clinicaltrials.gov) and the ISRCTN database (https://www.isrctn.com). Search terms included: “depression,” “completed,” “adult,” “interventional,” “with results,” and “imaging”. Our search terms were kept broad to be maximally inclusive, although this could result in a high exclusion rate. These search criteria were selected to identify completed studies with published results that have used imaging and pharmacological interventions in adult populations. Identified studies were assessed for their relevance in depression. An additional search was then conducted using the trial number for each study, in order to ensure that publications directly related to these trials but not listed in the clinical trials databases were also examined. All trials conducted using pharmacological compounds with antidepressant action that used fMRI (resting-state and/or task-based) to assess their efficacy and/or use in depression were included in this review. This selection process is outlined in Supplementary Data Sheet S1.

As a second-level search, the antidepressants used in the clinical trials identified in the first search were entered as search terms in PubMed (https://pubmed.ncbi.nlm.nih.gov). This was done to identify research studies relevant to depression that used fMRI (resting-state and/or task-based) to examine the effects of these compounds on brain activity and connectivity in healthy volunteers or patient groups but not in a clinical trial setting. Research studies that used compounds with antidepressant action solely as models for mental health disorders (for example, ketamine as a model for psychosis) without assessing the efficacy of these compounds in treating depression were excluded.

Trial selection and reporting of relevant studies followed the PRISMA guidelines for Systematic Review, as indicated in Supplementary Data Sheet S1. All publications were assessed for eligibility by the authors. The NbN3 nomenclature was followed to describe compounds with antidepressant action (Frazer and Blier, 2016).

### 2.2. Study classification

**Figure 1:**
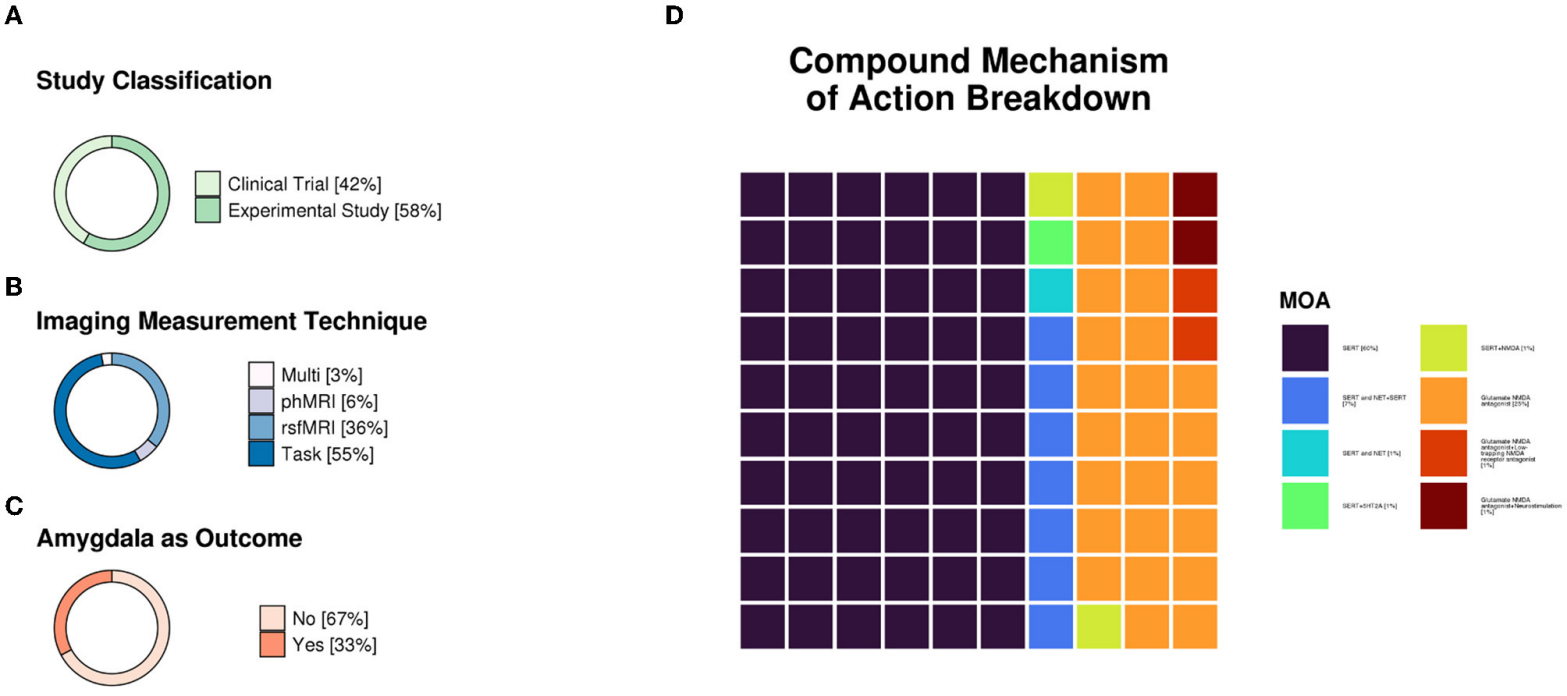
**(A)** Study classification distributions (n = 28 clinical trials and n = 39 experimental studies). **(B)** Imaging measurement technique distributions. Pharmacological functional magnetic resonance imaging (phfMRI), n = 4; resting-state functional MRI (rsfMRI), n = 24; task = data collected during stimulus presentation and/or response, n = 37; multi = two or more techniques used in one study (e.g., rsfMRI and task), n = 2. **(C)** Was the amygdala an outcome reported in the study (n = 22 ‘yes' and n = 45 ‘no'). **(D)** Compound mechanism of action breakdown. SERT, n = 40; SERT and NET+SERT, n = 5; SERT and NET, n = 1; SERT+H5T2A, n = 1; SERT+NMDA, n = 1; MDA antagonist, n = 17; NMDA antagonist+low-trapping NMDA receptor antagonist, n = 1; NMDA antagonist+neurostimulation, n = 1. Mechanisms of action were primarily referenced from Neuroscience Based Nomenclature (“NbN”; https://nbn2r.com/, https://www.sagepub.com/sites/default/files/nbn_glossary.pdf). “+” indicates that antidepressants with differing mechanisms of action were used in the study, whereas “and” indicates that a given compound has multiple theorized mechanisms of action. If multiple studies were reported in one paper, each study was counted separately. SERT, serotonin transporter; NET, norepinephrine transporter; 5-HT2, 5-hydroxytryptamine 2; NMDA, N-methyl-D-aspartate.

All studies fulfilling the search and selection criteria are presented in Tables 1, 2. Table 1 includes publications from studies selected using the clinical trial databases, and Table 2 includes research studies that investigated antidepressants identified from the clinical trials search. Studies were classified based on whether they used task-based or resting-state fMRI. A brief description of the recruited sample, methodology, main findings, and conclusions from each study are also included in the tables. Sample size and age, functional imaging technique, as well as the mechanisms of action of the compounds investigated in all included studies are summarized in Figure 1.

**Table 1:** Clinical trials investigating compounds with antidepressant action. Articles are listed by date of publication.

	**Article**	**Aim/Hypotheses**	**Experimental design**	**Rest/task-based fMRI**	**Study conclusions**
1	Sheline et al. (2001)	**Aim:** To use fMRI to demonstrate the role of the amygdala in non-conscious emotional processing before and after antidepressant treatment. **Hypotheses:** Depressed patients would have greater bilateral amygdala activation in response to masked fearful faces compared to HVs., and this would resolve with antidepressant treatment.	11 right-handed participants (6 women, mean age = 40.3) and 11 HVs. (6 women, mean age-39.8). Depressed patients were treated with sertraline. Clinical doses were titrated according to clinical response at subsequent bi-weekly visits and on average were ~100 mg. All participants were scanned at baseline and 8 weeks post-treatment.	Masked faces paradigm	Participants with depression presented with greater activation of the left amygdala in response to fearful faces, even when masked and unconsciously presented. The observed effect resolved with sertraline treatment.
2	Anand et al. (2005)	**Aim:** To use low frequency BOLD weighted temporal fluctuations to measure corticolimbic connectivity at rest and during emotional pictures **Hypotheses:** Antidepressant treatment would increase corticolimbic connectivity.	12 un-medicated unipolar depressed participants (9 female, mean age = 30) and 11 matched HVs. (8 female, mean age = 29). Participants received sertraline started at 50 mg which increased to 100 mg after 1 week; after 2 weekly visits sertraline was increased by 50 mg to a maximum of 200 mg. All participants were scanned at baseline and 6 weeks post-treatment.	Visual Picture Sequence rsMRI	Increased corticolimbic connectivity following treatment in depression was associated with increased emotion regulation.
3	Heller et al. (2009)^α^	**Aim:** To test whether depressed patients are unable to sustain activation in neural circuits relating to positive affect and reward. **Hypotheses:** Depressed patients would fail to sustain activity in the striatum when upregulating affect in response to positive stimuli. This would be confirmed by decreased NAcc activation and connectivity to the PFC.	27 individuals with depression (mean age = 31.5 and 19 HVs. (mean age = 31.8). Individuals with depression were randomized to two treatment groups: venlafaxine-ER (10 participants) or fluoxetine (17 individuals). All participants were scanned at baseline and after treatment	Emotional Regulation Task.	Anhedonia in depressed patients reflected the inability to sustain engagement of structures involved in positive affect and reward as demonstrated by decreased NAcc activation.
4	Stoy et al. (2012)	**Aim:** To examine whether escitalopram could modify VS activity during anticipation of gain and loss in depressed patients to assess whether dysfunction in the VS is associated with symptom severity and anhedonia **Hypotheses:** Reduced VS activation would be observed in unmedicated depressed patients during anticipation of gain and loss. Successful treatment with escitalopram would diminish VS alterations.	15 unmedicated depressed adults (5 female, mean age = 41.9) and 15 HVs. (5 female, mean age = 39.5). Participants received 5 mg of escitalopram for 3 days and an additional 5 mg every 3 days until a therapeutically effective dose was reached. Scans were acquired at baseline and 6 weeks post-treatment.	Monetary Incentive Delay Task	Unmedicated depressed patients had hyporeactivity of the VS during anticipation of positive and negative monetary incentives relative to HVs. prior to, but not following, treatment with escitalopram.
5	Heller et al. (2013a)^α^	**Aim:** To examine whether changes induced by treatment in sustained NAcc activity and fronto-striatal connectivity during positive affect regulation were associated with subsequent gains in positive affect **Hypotheses:** Over 2 months of treatment, positive affect would increase and negative affect would decrease. Changes in sustained NAcc activity and fronto-striatal connectivity, brought on by treatment, would correlate with positive affect changes.	27 individuals with depression (mean age = 31.5) and 19 HVs. (mean age = 31.8). Individuals with depression were randomized to two treatment groups: venlafaxine-ER (10 participants) or fluoxetine (17 individuals). Participants were scanned at baseline and after treatment.	Emotional Regulation Task	Positive day-to-day emotion was related to sustained engagement of fronto-striatal circuitry induced by treatmen.
6	Heller et al. (2013b)^α^	**Aim:** To examine the neurobiological effects of treatment. **Hypotheses:** Changes in PFC and amygdala activity would correlate with change in depression scores.	27 individuals with depression (mean age = 31.5) and 19 HVs. (mean age = 31.8). Those with depression were randomized to two treatment groups: venlafaxine-ER (n = 10) or fluoxetine (n = 17). Participants were scanned at baseline and after treatment.	Emotional Regulation Task	Changes in depression severity over 6 months was correlated with PFC engagement during negative affect regulation.
7	Fu et al. (2015)	**Aim:** Multimodal investigation of the functional and structural neuroanatomy of depression in a prospective, longitudinal design in individuals experiencing a major depressive episode while receiving treatment and HVs **Hypotheses:** Treatment would be associated with normalization of ACC and amygdala activation in response to sad faces in depressed patients compared to HVs.	32 medication-free depressed patients (13 female, mean age = 40.2) experiencing an acute depressive episode and 25 HVs. (13 female, mean age = 38.8). Patients received treatment of duloxetine (60 mg daily) for 12 weeks with the option to increase to 120 mg daily after 8 weeks. Scans were acquired at weeks 0, 1, 8, and 12 post-treatment.	Emotional face processing paradigm; negative attentional bias (Stroop) paradigm; rsMRI	Effects in PCC demonstrated in response to task designed to engage negative attentional bias in depression. Time-dependent changes were observed in the DMN in depressed patients over the course of treatment. Baseline resting-state connectivity within the orbitofrontal component of the DMN significantly predicted treatment response.
8	Downey et al. (2016)	**Aim:** To investigate whether a single dose of lanicemine would reproduce previously reported decreases in sgACC activity elicited by ketamine **Hypotheses:** Ketamine and lanicemine would have similar effects on neural systems implicated in depression.	20 depressed patients (12 females, mean age = 26.7) in the lanicemine group, 21 depressed patients (13 female, mean age = 27.1) in the ketamine group, 19 depressed patients (11 female, mean age = 25.7) in the placebo group. Randomized, double-blind, placebo-controlled, parallel group design. Scans were acquired before and during drug administration.	phfMRI	Ketamine and lanicemine gradually increased BOLD signal in the sgACC and rostral ACC.
9	Abdallah et al. (2017)	**Aim:** To investigate PFC activity in patients with depression and how PFC activity would change in response to treatments for depression **Hypothesis:** Depressed patients experiencing a current major depressive episode will show reduced PFC GBC. Mood normalization following ketamine treatment would parallel normalization in functional connectivity.	18 depressed patients (8 female, mean age = 43) and 22 HVs. (12 female, mean age = 39). Ketamine was administered intravenously. Scans were acquired at baseline and 24 h post-ketamine infusion.	rsMRI	Ketamine responders showed increased GBC in the lateral PFC, caudate, and insula. Follow-up seed-based analyses illustrated a pattern of dysconnectivity between the PFC/subcortex and the rest of the brain in individuals with depression that appeared to normalize post-ketamine. The extent of the functional dysconnectivity identified in depression and the swift and robust normalization following treatment suggest that GBC may serve as a treatment response biomarker for the development of rapid-acting antidepressants.
10	Evans et al. (2018)^λ^	**Aim**: To examine connectivity changes in the DMN 48h and 2 weeks after ketamine **Hypothesis:** DMN differences between HVs. and individuals with depression would be reduced after ketamine administration, particularly in regions associated with the SAL and CEN.	33 depressed patients (20 female, mean age = 36) and 25 HVs. (12 female, mean age = 33) Placebo-controlled, crossover design of intravenous ketamine infusion. Scans were acquired at baseline and 48 h post-ketamine infusion.	rsMRI	Connectivity changes in the insula in individuals with depression suggest that ketamine may normalize the interaction between the DMN and salience networks, supporting the triple network dysfunction model of depression.
11	Chen et al. (2019)	**Aim:** To determine the effects of ketamine on the functional connectivity of PFC-related circuits in patients with TRD **Hypothesis:** Ketamine would produce critical increases and decreases in PFC-related circuits, and these changes would correlate with alleviation of symptoms.	48 patients with TRD received 0.5 mg/kg of ketamine (11 female, mean age = 43.3), 0.2 mg/kg of ketamine (11 female, mean age = 44.4) or placebo (13 female, mean age = 49.9). Randomized, placebo-controlled, double-blind design. Scans were acquired at baseline and on Day 3 post-ketamine infusion.	rsMRI	Connectivity changes in PFC-related circuits were associated with decreased clinical symptoms and suicidal ideation in both the standard and low-dose groups. PFC-related circuit modulation was crucial to the antidepressant and antisuicidal effects of ketamine.
12	Kraus et al. (2020)^λ^	**Aim:** To independently replicate the finding of disrupted GBC in individuals with depression and examine the specific effects of preprocessing strategies on GBC. **Hypothesis:** Ketamine would significantly reduce GBC.	33 depressed patients (20 female, mean age = 36) and 25 HVs. (12 female, mean age = 33). Placebo-controlled, crossover design of intravenous ketamine infusion. Scans were acquired at baseline and 48 h post-ketamine.	rsMRI	Reduced GBC was observed in depressed patients at baseline in the anterior and medial cingulate cortices, as well as the PFC. Ketamine did not produce any significant changes in connectivity.
15	Tozzi et al. (2020)	**Aim:** To investigate whether pre-treatment activation and coupling during cognitive inhibition would predict clinical response following 8 weeks of acute treatment with escitalopram, sertraline, or venlafaxine-XR, and to identify predictors and correlates of response for each treatment. **Hypotheses:** Regions associated with treatment response for each medication would include the dlPFC, dorsal parietal cortex, and precentral gyrus. To investigate whether pre-treatment activation and coupling during cognitive inhibition would predict clinical response following 8 weeks of acute treatment with escitalopram, sertraline, or venlafaxine-XR, and to identify predictors and correlates of response for each treatment.	59 HVs. (29 female, mean age = 30.37), 59 depressed patients (29 female, mean age = 30.4) who received escitalopram, 19 depressed patients (7 female, mean age = 37.7) who received sertraline, and 21 depressed patients (14 female, mean age = 35.5) who received venlafaxine-XR. Randomized design. Scans were acquired at baseline and 8 weeks after treatment.	Go/No Go paradigm	In depressed patients, connectivity of the cognitive control circuit—including the insula and the post central and temporal gyrus—during a response inhibition task predicted response to antidepressant treatment and correlated with symptom improvement over time.
17	Loureiro et al. (2020)	**Aim:** To examine the neuronal underpinnings of emotional processing within and across patients receiving ketamine or ECT) **Hypotheses:** Reductions in amygdala activity in response to negative stimuli would follow ECT treatment.	32 HVs. (19 female, mean age = 34.5) and 44 individuals with TRD who received either ketamine (27 patients, 11 female, mean age = 37.3) or ECT (17 patients, 12 female, mean age = 36.8). Scans were acquired at baseline (pre-treatment) and 24 h−72 h after the last ketamine infusion or within a week of completing the ECT index series.	Emotional face processing paradigm	Decreased amygdala reactivity was observed after both ECT and ketamine for both positive and negative emotional face processing. Subtler treatment-specific changes occurred in the larger functional network.
18	Sahib et al. (2020a)^θ^	**Aim:** To investigate the functional connectome of the brain at rest after acute and serial ketamine infusions in order to establish whether treatment-related FC changes normalize toward control values and to identify correlates of response for depressed patients who remitted. **Hypotheses:** Functional connectivity changes in the SN would characterize treatment response.	40 HVs. (24 female, mean age = 32) and 61 individuals with TRD (19 female, mean age = 38) received a series of 4 ketamine treatments. Scans were acquired at baseline, 24 h after the first ketamine infusion, and 24 h or 72 h after the last ketamine infusion. HVs. had one scan.	rsMRI	Significant differences in resting-state functional connectivity were observed between HVs. and depressed patients at baseline in the SMN and between the association network and DMN. These disruptions trended toward healthy patterns following the ketamine infusions. Cortico-striatal-cerebellar loops that encompass the SN appeared to be a potential biomarker for ketamine treatment.
19	Sahib et al. (2020b)^θ^	**Aim:** To examine treatment-related changes in the inhibitory control network following acute and serial ketamine treatment in TRD. **Hypotheses:** Response-inhibitory activity would differentiate remitters from non-remitters, and regional brain activity would differ following ketamine treatment.	40 HVs. (24 female, mean age = 32) and 61 individuals with TRD (19 female, mean age = 38) received a series of 4 ketamine treatments. Scans were acquired at baseline, 24 h after the first ketamine infusion, and 24 h or 72 h after the last ketamine infusion. HVs. had one scan.	Go/No Go paradigm	Significant decreases in brain activation in the inhibitory control network followed serial ketamine treatment. Changes in the sensorimotor area were related to reductions in depressive symptoms, suggesting that modulation of this network may play a role in therapeutic response.
13	Mkrtchian et al. (2021)^λ^	**Aim:** To investigate ketamine's effects on fronto-striatal connectivity 48 h and 2 weeks after its administration. **Hypothesis:** Ketamine would increase functional connectivity within the fronto-striatal circuitry of TRD participants but decrease connectivity in HVs. These effects would be associated with ketamine-induced changes in inflammatory response.	33 depressed patients (20 female, mean age = 36) and 25 HVs. (12 female, mean age = 33). Placebo-controlled, crossover design of intravenous ketamine infusion. Scans were acquired at baseline and 48 h post-ketamine.	rsMRI	Ketamine normalized fronto-striatal connectivity in patients with depression 48 h post-administration. This normalization effect was associated with both acute and sustained improvement of the antidepressant response.
14	Vasavada et al. (2021)^θ^	**Aim:** To investigate the effect of serial ketamine treatments in the connectivity between limbic structures and the RSNs. **Hypothesis:** Functional connectivity between the amygdala and/or hippocampus and cortical RSNs would be deficient in individuals with TRD and restored by ketamine.	40 HVs. (24 female, mean age = 32) and 61 individuals with TRD (19 female, mean age = 38) received a series of 4 ketamine treatments. Scans were acquired at baseline, 24 h after the first ketamine infusion, and 24 h or 72 h after the last ketamine infusion. HVs. had one scan.	rsMRI	Increases in functional connectivity between limbic regions and the CEN suggest that ketamine might work by restoring top-down control of emotional processing. Functional connectivity between the left amygdala and the SAL suggests that ketamine might also restore the dysconnectivity between those areas observed in depression.
16	Abdallah et al. (2021)	**Aim:** To identify the effects of ketamine on brain circuitry and networks in order to determine biomarkers of treatment response. **Hypotheses:** Cortical functional connectivity changes with ketamine would be reproducible in different cohorts; these connectivity changes would predict treatment response to antidepressant treatment (sertraline).	265 participants collected across four different studies. Study 1: 25 HVs. (6 female, mean age = 30); Study 2: 22 HVs. (8 female, mean age = 29); Study 3: 18 male HVs. (mean age = 28); Study 4: 200 unmedicated depressed patients (133 female, mean age = 38).	rsfMRI	A robust and reproducible ketamine connectome fingerprint was identified that predicted response to sertraline, suggesting that it is related to the mechanism of action of antidepressants.
20	Rivas-Grajales et al. (2021)	**Aim:** To examine possible changes in habenula connectivity in patients with TRD who received a single subanesthetic dose of ketamine. **Hypotheses:** Improvement of depression severity would be associated with greater connectivity between the habenula and prefrontal areas. Improvement of depressive symptoms would be associated with reduced connectivity of the habenula and ventral tegmental area.	35 individuals with TRD (16 female, mean age = 42.2). Randomized design where patients were assigned to a ketamine or midazolam arm (only ketamine is reported here). Scans were acquired at baseline, at 24 h prior to infusion, and at 24 h following the infusion.	rsMRI	Increased functional connectivity between the habenula and PFC may be involved in ketamine's antidepressant effects in individuals with TRD.
21	Siegel et al. (2021)	**Aim:** To replicate previous study findings showing the feasibility of ketamine as a treatment for depression and treatment response to ketamine, as well as to identify potential correlates of ketamine's antidepressant effects. **Hypotheses:** Responders to ketamine would demonstrate response-dependent connectivity decreases in the sgACC and DMN.	23 TRD patients (10 female, mean age = 40). Open-label study with a continuous 96 h infusion of intravenous ketamine, from 0.15 mg/kg/h and titrated to 0.6 mg/kg/h. Participants started on clonidine and discontinued upon infusion completion.	rsMRI	DMN hypoconnectivity and sgACC hyperactivity normalized after the continuous ketamine infusion, and the observed effects were similar to repetitive short infusions. Increased connectivity in the limbic system, and the hippocampus specifically, were also observed after ketamine.
22	Nakamura et al. (2021)	**Aim:** To examine the utility of rsfMRI for predicting treatment response to ketamine in TRD. **Hypotheses:** Connectivity within the affective network and the DMN would be the neural basis of treatment response heterogeneity in TRD.	15 TRD participants (9 female, mean age = 45.9) received a ketamine infusion; 11 of these participants received four infusions. Eight were classified as full responders. Scans were acquired 16 h before the first ketamine infusion and 6–24 h after the last ketamine infusion.	rsMRI	Exploratory analyses identified a cluster of significant baseline resting-state functional connectivity differences associating ketamine response and activation of the amygdala and subgenual anterior cingulate gyrus in the right hemisphere. This altered functional connectivity reflected antidepressant response to ketamine at baseline. rsMRI also appeared to be useful in predicting treatment response.
23	Loureiro et al. (2021)^θ^	**Aim:** To investigate how single, serial ketamine infusions would modulate cerebro-cerebellar networks; how these circuitries relate to treatment remission; and to examine if these changes are associated with secondary clinical outcomes. **Hypotheses:** Connectivity between the cerebellum, SMN, and FPN would be disrupted in patients with TRD prior to treatment and might distinguish treatment remitters from non-remitters.	40 HVs. (24 female, mean age = 32) and 61 individuals with TRD (19 female, mean age = 38) received a series of 4 ketamine treatments. Scans were acquired at baseline, 24 h after the first ketamine infusion, and 24 h or 72 h after the last ketamine infusion. HVs. had one scan.	Go/No Go paradigm	Significantly decreased connectivity following ketamine between the cerebellum and FPN and SMN networks (only in remitters). Ketamine also modulated connectivity between the cerebellum and cortical networks such as the FPN, SMN, and SN, suggesting that it may regulate function through cerebellar-cortico loops.
24	Kilpatrick et al. (2022)	**Aim:** To evaluate the relationship between changes in within-DMN connectivity and improvement in depression following 3 months of treatment. **Hypotheses:** Escitalopram-memantine treatment would show more pronounced changes in within-network DMN connectivity, associated with greater symptom improvement.	Twenty six patients with depression received combined escitalopram-memantine (13 patients, 7 female, mean age = 53.8) vs. escitalopram-placebo (13 patients, 8 female, mean age = 61.5) (10 mg escitalopram for 4 weeks, then 20 mg escitalopram for 4 weeks). The daily dose of memantine or matched placebo was gradually increased from 5mg to 20 mg over 4 weeks. Randomized, placebo-controlled clinical trial. Scans were acquired at baseline and at 3 months follow-up.	rsMRI	Escitalopram/memantine combined therapy was more effective than escitalopram and placebo in improving depressive symptoms after 3 months of treatment. Increased within-network connectivity of posterior and lateral nodes of the DMN correlated with greater improvement in depression severity.
25	Daws et al. (2022)	**Aim:** To better understand psilocybin's antidepressant action. **Hypotheses:** The well-replicated finding of brain network disintegration and desegregation under psychedelics will be apparent subacutely in post-treatment rsfMRI data. This effect will relate to improved depression outcomes and will not be observed after escitalopram administration.	Study1: Open-label trial for 16 patients (4 females, mean age = 42.7) with TRD in a single-arm clinical trial. Baseline rsfMRI followed by fixed-order “low” (10 mg and “high” (25 mg) psilocybin with doses separated by 1 week. A second fMRI was conducted 1 day after the second drug dose. Study 2: Double-blind, placebo-controlled trial. 43 depressed patients were allocated to the psilocybin arm (22 patients, 8 female, mean age = 44.5) or the escitalopram arm (21 patients, 6 female, mean age = 40.9). Baseline fMRI followed by 25 mg psilocybin or 1 mg psilocybin. The escitalopram arm involved 3 weeks of 10 mg daily escitalopram, followed by 3 weeks of 20 mg of escitalopram daily.	rsMRI	In both trials, decreased brain modularity was observed and correlated with improvements in depressive symptomatology. This antidepressant action may be specific to psilocybin therapy. Increased global brain network integration accompanied the antidepressant efficacy of psilocybin therapy.

Greek letters (α, λ, θ) indicate that the same samples were used across papers.

ACC, anterior cingulate cortex; BOLD, blood oxygen level dependent; CEN, central executive network; dlPFC, dorsolateral prefrontal cortex; DMN, default mode network; ECT, electroconvulsive therapy; FC, functional connectivity; FPN, frontoparietal network; GBC, global brain connectivity; HV, healthy volunteer; NAcc, nucleus accumbens; PCC, posterior cingulate cortex; PFC, prefrontal cortex; rsfMRI, resting-state functional magnetic resonance imaging; RSN, resting state network; SAL, salience network; sgACC, subgenual anterior cingulate cortex; SMN, sensorimotor network; TRD, treatment-resistant depression; VS, ventral striatum; WM, working memory.

**Table 2:** Research studies investigating compounds with antidepressant action. Articles are listed by date of publication.

	**Article**	**Aim/Hypotheses**	**Experimental design**	**Rest/task-based fMRI**	**Summary**
1	Fu et al. (2004)^μ^	**Aim:** To examine whether individuals with depression differ from HVs. in activation of negative affect processing systems, given that individuals with depression typically show antidepressant treatment-related changes in activation of such systems. **Hypotheses:** Increased amygdala response to sad faces at baseline would be attenuated after treatment while depressive symptom reductions would be associated with ACC activation.	Nineteen participants with depression (11 female, mean age = 42.8) and 19 HVs. (13 women, mean age = 43.2). Parallel group, repeated measures design with depressed patients receiving fluoxetine, 20 mg daily for 8 weeks. Scans were acquired at baseline, 2 weeks, and 8 weeks post-treatment.	Implicit sad facial affect recognition paradigm	Over the course of treatment, abnormally exaggerated response to sad faces at baseline significantly reduced in response to fluoxetine (including reduced activation of the left amygdala). Changes associated with symptomatic improvement suggest that fMRI may be a useful marker of antidepressant treatment response.
2	Del-Ben et al. (2005)^ξ^	**Aim:** To determine the acute effects of citalopram on brain activation during cognitive activation tasks. **Hypotheses:** In the Go/NoGo paradigm, NoGo relative to Go blocks would be associated with increased BOLD response in lateral OFC, anterior cingulate, right middle frontal gyrus, and dlPFC. In the Loss/NoLoss paradigm, the main effect of Loss compared to NoLoss would be associated with neural responses in the lateral OFC and hippocampus. In the covert aversive face emotion recognition paradigm, increased BOLD response would be observed in the amygdala, fusiform gyrus, OFC, and anterior cingulate gyrus.	Twelve male HVs. (mean age = 24.7). Randomized, placebo-controlled, crossover design of placebo or intravenous citalopram. Scans were acquired during the drug infusion.	Go/NoGo paradigm Loss/NoLoss paradigm Covert aversive face emotion recognition paradigm	Pre-treatment with citalopram enhanced lateral OFC response to the NoGo and NoLoss conditions. The right amygdala response to aversive faces was also attenuated by citalopram. The technique of combining drug challenge with fMRI may be promising for investigating human psychiatric disorders.
3	McKie et al. (2005)^ξ^	**Aim:** To examine the effects of intravenous citalopram as a pharmacological challenge. **Hypotheses**: Citalopram would affect BOLD response in brain areas known to be implicated in depression and in response to antidepressants.	Twelve male HVs. (mean age = 24.7). Randomized, placebo-controlled, crossover design of placebo or intravenous citalopram. Scans were acquired during the drug infusion.	phfMRI	Citalopram infusion activated brain areas implicated in depression, including the subgenual cingulate gyrus, the amygdala, the caudate, and the ventral frontal cortex. Direct phfMRI using intravenous citalopram opens new ways of investigating 5-HT mechanisms in depression and its treatment.
4	Harmer et al. (2006)	**Aim:** To investigate the effects of short term SSRI administration on amygdala response in HVs. **Hypotheses:** Citalopram administration would decrease amygdala responses to masked fearful facial expressions.	Twenty four right-handed HVs. (14 female, mean age = 26). Randomized, double-blind design of either 20mg of citalopram or placebo every day for 7 days. Scans were acquired at baseline and 7 days post-citalopram administration.	Emotional masked faces paradigm	Citalopram administration for 7 days significantly attenuated amygdala response to fearful faces compared to placebo. These results indicate a direct and dissociable effect of SSRI administration on the processing of subliminal emotional stimuli within the amygdala that was not mediated by symptom remission in depression.
5	Rose et al. (2006)	**Aim:** To investigate whether a short course of an SSRI given at a typical clinical dose would affect brain function measured using BOLD fMRI during performance of a working memory task in HVs. **Hypotheses:** Escitalopram would facilitate working memory performance and alter brain metabolism in regions commonly associated with the affective profile of treatment-responsive depressed patients.	Ten HVs. (3 female, mean age = 24.6). Participants received 10 mg/day escitalopram for 7 days. Scans were acquired at baseline and 7 days post-escitalopram administration.	N-back task	Escitalopram significantly altered activation in the thalamus, hypothalamus, and amygdala while reducing activity in the cingulate cortex. Task performance and cognitive function were not affected.
6	Chen et al. (2007)	**Aim:** To identify a predictor for antidepressant treatment response. **Hypotheses:** Baseline symptom severity and treatment response would both be associated with MRI measures of ACC function and structure. Depressed patients with reduced gray matter volume or task-related activation in the ACC would have more severe symptoms at baseline and/or slower rates of treatment response.	Seventeen depressed participants (12 female, mean age = 44) received antidepressant treatment with oral fluoxetine hydrochloride, 2 mg/day for 8 weeks. Scans were acquired at baseline.	Facial affect processing paradigm	Faster rates of symptom improvement were strongly associated with greater gray matter volume in the ACC, insula, and right temporo-parietal cortex. Baseline symptom severity was negatively correlated with greater gray matter volume in the dorsal prefrontal and anterior midcingulate regions anatomically distinct from the pgACC and sgACC, predicting treatment response.
7	Fu et al. (2007)^μ^	**Aim:** To examine the brain areas involved in happy facial expression processing and how activation of these brain areas changes in depression and after treatment. **Hypotheses:** Compared to HVs., individuals with depression will display less neural response to happy faces in the basal ganglia and in limbic regions (caudate, putamen, and hippocampal regions).	Nineteen medication-free participants with depression (15 female, mean age = 42.8) and 19 HVs. (13 women, mean age = 43.2). Parallel group, repeated-measures design where depressed patients received 20 mg of fluoxetine daily for 8 weeks. Scans were acquired at baseline, 2 weeks and 8 weeks post-drug administration.	Implicit Recognition of Happy Facial Affect paradigm	Depressed patients exhibited attenuated responses in limbic-subcortical and extrastriate visual regions, in response to happy faces. These attenuated responses were increased post-treatment and associated with symptom improvement.
8	Anderson et al. (2007)^ξ^	**Aim:** To investigate the effects of acute serotonergic modulation on neural responses to specific negative face emotions. **Hypotheses:** Compared with neutral faces, all three emotional faces would cause increased BOLD signal in the amygdala, fusiform gyrus, and lateral OFC. Angry faces would activate the anterior cingulate gyrus and disgusted faces would activate the insular cortex and putamen. Citalopram would attenuate these responses.	Twelve male HVs. (mean age = 24.7). Randomized, placebo-controlled, crossover design of placebo or intravenous citalopram. Scans were acquired during the drug infusion.	Covert emotional recognition task	Citalopram did not affect activation to angry faces, but it attenuated right amygdala activation to fear and left amygdala activation to disgust.
9	Wingen et al. (2008)^π^	**Aim:** To define the brain regions involved in performance during the Mackworth Clock Test. To assess the effects of increased serotonin levels in HVs. on sustained attention and on the brain areas that underlie it. **Hypotheses:** The right parietal and frontal brain areas would be activated during the Mackworth Clock Test.	Ten HVs. (5 female, mean age = 26.3). Double-blind, placebo-controlled, crossover design where treatment consisted of 20mg escitalopram and placebo administered on 2 different test days separated by a washout period of at least 7 days. Scans were acquired 4 h post-drug or post-placebo administration.	Mackworth Clock Test	Serotonin stimulation may impair sustained attention by modulating selective brain areas, including prefrontal areas and parts of the basal ganglia, which are possibly involved in a subcortical network for sustained attention.
10	Windischberger et al. (2010)	**Aim:** To compare the effect of two SSRIs on brain activation by examining phfMRI during facial emotion processing. **Hypotheses:** Citalopram and escitalopram administration would reduce the responsivity of regions involved in emotional processing compared to placebo. Escitalopram and citalopram treatment would induce significant differences in brain activation.	Twenty four HVs. (6 female, mean age = 24.8). Randomized, placebo-controlled design of 20 mg citalopram per day for 10 days, 10 mg escitalopram per day for 10 days, or placebo. Scans were acquired following 10 days of citalopram, escitalopram, or placebo.	Alternating blocks of facial expression discrimination and shape discrimination	Task performance was not altered by medication. Escitalopram and citalopram affected the processing of emotional content, as evidenced by attenuation in reactivity of the right amygdala and left parahippocampal cortex in response to emotional stimuli relative to neutral stimuli.
11	Matthews et al. (2010)^ρ^	**Aim:** To investigate the degree to which SSRI administration would affect activity in the anterior and posterior medial cortex, including the ACC and PCC, during self-referential processing. **Hypotheses:** 3 weeks of escitalopram administration would decrease functional activity in the medial cortex during self-referential processing	Eighteen female HVs. (mean age = 22.3). Randomized, placebo-controlled, crossover design where escitalopram (starting at 5 mg/day) or placebo were administered for 3 weeks. Scans were acquired at the end of the 3-week period of drug administration.	Self-referential processing task with three trial types: self-evaluation, other evaluation, word evaluation	3-week administration of the SSRI escitalopram was associated with increased positive self-endorsement and with attenuation of posterior medial cortex activity during self-referential processing in HVs.
12	Rosenblau et al. (2012)	**Aim:** To assess neural activation during anticipation of the response to unpleasant stimuli in order to examine the neural correlates of impaired processing of emotions in depression. **Hypotheses:** Depressed individuals would display greater amygdala activation during anticipation of and response to negative stimuli compared to HVs. as well as displaying reduced activation in prefrontal regions. These dysfunctions would be normalized following treatment with escitalopram.	Twelve un-medicated individuals with depression (7 female, mean age = 43.5) and 12 HVs. (7 female, mean age = 45.8). Depressed patients received 5 mg escitalopram for the first 3 days and an additional 5 mg every 3 days until a therapeutically effective dose was reached. Scans were acquired at baseline and at 6–8 weeks post-treatment.	Affective pictures paradigm	Increased amygdala and prefrontal activation during anticipation of negative images was observed in depressed patients vs. HVs. Aberrant processing was resolved following treatment with escitalopram.
13	Godlewska et al. (2012)	**Aim:** To assess whether escitalopram treatment in individuals with depression would remediate neural changes in emotional bias. **Hypotheses:** Compared to placebo, escitalopram treatment would attenuate amygdala response to fearful faces in the absence of clinically important changes in mood ratings.	Forty five patients with depression (21 were treated with placebo (11 female; mean age = 31.1) and 21 were treated with escitalopram [12 female, mean age = 32.1)] and 18 HVs. (13 female, mean age = 33.9). Double-blind, parallel group, placebo-controlled study where participants received 10 mg of escitalopram or placebo for 7 days. Scans were acquired at baseline and immediately after treatment.	Gender discrimination task	Amygdala responses to fearful faces were significantly lower in depressed patients treated with escitalopram for 7 days compared to those receiving placebo. These differences were not seen in clinical ratings.
14	Alexopoulos et al. (2012)	**Aim:** To assess whether functional connectivity at rest within the CCN and DMN characterizes late-life depression and predicts antidepressant treatment. **Hypotheses:** Low rsFC within the CCN and high rsFC within the DMN would distinguish depressed older adults from older HVs. Lower rsFC of the CCN during depressive episodes would predict persistence of symptoms and signs during treatment.	Sixteen older adults with depression (mean age = 69) and 10 HVs. (mean age = 68.6). Single-blind placebo design of a 2-week placebo phase and then 12 weeks of escitalopram beginning at 10 mg/day for one week and increasing to 20 mg. Scans were acquired at the end of the placebo phase for depressed patients and upon study entry for the HVs.	rsfMRI	Low rsFC in the CCN and high rsFC within the DMN characterized late-life depression. Identifying network abnormalities underlying late-life depression can provide novel targets for testing new somatic and psychosocial treatments.
15	Miller et al. (2013)	**Aim:** To explore the relationship between negative words and treatment outcomes over the course of escitalopram treatment for depression. **Hypotheses:** Lower activity in the sgACC in response to negative words would be associated with better treatment outcomes.	Fifteen un-medicated participants with depression treated for 8 weeks. The dose was 10 mg escitalopram, increased to 20 mg after 4 weeks for non-responders. Scans were acquired at baseline.	Negative words task	Clusters in the left paracingulate gyrus, globus pallidus, and post-central gyrus were maximally predictive of treatment outcome. Participants whose activity in the sgACC scaled positively with emotional valence had better treatment outcomes.
16	Wang et al. (2014)	**Aim:** To assess cross-sectional brain function in drug-naïve patients with depression. **Hypotheses:** 8 weeks of escitalopram would modulate activity in the fronto-limbic circuit, including the dlPFC, thalamus, and insula.	Fourteen patients with depression (5 female, mean age = 32.9) and 14 HVs. (5 female, mean age = 34.1). Patients received escitalopram treatment (10 mg/day) for 8 weeks. Scans were acquired at baseline, and patients were also scanned at the end of the treatment period.	rsfMRI	Successful treatment with escitalopram was associated with modulation of resting-state brain activities in regions within the fronto-limbic mood circuit of depressed patients.
17	van de Ven et al. (2013)^π^	**Aim:** To investigate the intrinsic functional connectivity of the DMN as a function of activity of the serotonergic system through the administration of escitalopram. **Hypotheses:** Compared to placebo, escitalopram would decrease intrinsic FC in the DMN	Ten HVs. (5 female, mean age = 26.3). Double-blind, placebo-controlled, crossover design where treatment consisted of 20 mg escitalopram and placebo administered on 2 different test days separated by a washout period of at least 7 days. Scans were acquired 4 h post-drug or post-placebo administration.	rsfMRI	In HVs., escitalopram decreased the FC of several regions within the DMN. Resting-state phfMRI is a valuable neuroimaging tool to further investigate the intrinsic functional architecture of the brain.
18	Drueke et al. (2013)	**Aim:** To verify response inhibition and re-engagement using the stop-change paradigm under acute serotonergic inhibition. **Hypotheses:** Performance in the stop-change task would not be affected by 5-HT modulation.	Fourteen male HVs. Randomized, double-blind, placebo-controlled design where they received 10 mg escitalopram and placebo. Scans were acquired 3 h post-drug or post-placebo administration.	Stop-change paradigm	Escitalopram did not affect inhibitory performance but was associated with altered brain activation patterns compared to placebo. Results support the influence of 5-HT on response inhibition.
19	Outhred et al. (2014)^ϕ^	**Aim:** To explore the impact of escitalopram during processing of positive, negative, and neutrally emotional images. **Hypotheses:** Escitalopram would produce greater left amygdala activation in response to positive images and a decreased response to negative images.	Thirty six right-handed female HVs. (mean age = 25). Randomized, double-blind, placebo-controlled study where participants received 20 mg oral escitalopram or placebo. Scans were acquired 4 h after drug administration.	Emotional processing task	Amygdala response during positive and neutral stimuli increased while response to negative stimuli decreased under the escitalopram condition.
20	Wang et al. (2014)^χ^	**Aim:** To use fALFF to examine the effect of treatment with escitalopram on whole-brain spontaneous activity in patients with depression. **Hypotheses:** Escitalopram would modulate brain regions involved in emotional processing and regulation.	Thirty six first-episode, drug-naïve, non-comorbid depressed patients (11 female, mean age = 34.6) and 32 HVs. (11 female, mean age = 33.3). Depressed patients received 8 weeks of escitalopram 20 mg (*n =* 15), 15 mg (*n =* 4), or 10 mg (*n =* 1). Scans were acquired at baseline, and depressed patients were scanned at 8 weeks post treatment.	rsfMRI	Treatment-related changes in fALFF were found in several emotion-related brain regions, including the PFC and left putamen. fALFF values in these brain regions were significantly increased in depressed patients compared to HVs. at baseline and were reduced following treatment.
21	Chen et al. (2014)	**Aim:** To analyze the relative differences in neural activity between the right and left amygdalae in currently depressed patients during exposure to emotional stimuli under pre- and post-test (escitalopram) conditions. **Hypotheses:** No specific hypotheses stated. An exploratory approach was followed to examine amygdala activations.	Twenty un-medicated participants with depression (mean age = 45.7). 6 weeks of escitalopram treatment. Scans were acquired at baseline and at the end of the treatment period.	Emotional processing paradigm	Changes in BOLD signal in the amygdala following treatment with escitalopram suggests that this may be one way to understand depression's biological mechanisms. fMRI may be useful for evaluating the clinical manifestations of depression.
22	Williams et al. (2015)^ψ^	**Aim:** To examine whether amygdala activation probed by emotion stimuli is a general or differential predictor of response to 3 commonly prescribed antidepressants (SSRIs and SNRIs). **Hypotheses:** Pre-treatment amygdala activity would be a general predictor of treatment response and could differentially predict response by medication class.	Eighty participants with depression (40 female, mean age = 32.8) and 34 matched HVs. (16 female, mean age = 31.5). Depressed patients were randomized to 3 treatment groups: escitalopram (SSRI), sertraline (SSRI), or venlafaxine (SNRI) and were treated for 3 weeks. Scans were acquired at baseline, and depressed patients were also scanned at the end of the treatment period.	Facial emotion paradigm	Pre-treatment levels of amygdala activation to subliminally processed facial expressions that signal reward and threat generally predicted which patients would respond to antidepressants. Functional imaging biomarkers of amygdala activity may ultimately help tailor treatment selection according to which patients are generally likely to respond to SSRIs and which patients are specifically unlikely to respond to SNRIs.
23	Outhred et al. (2015)^ϕ^	**Aim:** To understand if treatment facilitates neural activity while participants are appraising and reappraising emotional stimuli. **Hypotheses:** Decreased left amygdala activity would be associated with increased right IFG activity. Left amygdala-right IFG functional connectivity changes would be reflected in behavioral responses.	Thirty six right-handed female HVs. (mean age = 25). Randomized, double-blind, placebo-controlled study where participants received 20 mg oral escitalopram or placebo. Scans were acquired 4 h after drug administration.	Emotion regulation task with “watch” and “think objectively” trials	A single dose of escitalopram facilitated inhibitory effects of reappraisal of emotional stimuli, at least in part, by modulating FC between the left amygdala and right IFG (in addition to other regions involved in emotion regulation).
24	Gyurak et al. (2016)^ψ^	**Aim:** To examine whether cognitive functioning, as assessed through neural activation prior to treatment, could predict remission and response with different types of antidepressant medications. **Hypotheses:** Neural activation during one or all 3 cognitive task probes would predict antidepressant response, and these signals would interact with medication type. Neural activation pre-treatment would differ at baseline between groups, and activation of the predictive regions would change as a function of treatment.	Eighty participants with depression (40 female, mean age = 32.8) and 34 matched HVs. (16 female, mean age = 31.5). Depressed patients were randomized to 3 treatment groups: escitalopram (SSRI), sertraline (SSRI), or venlafaxine (SNRI) and were treated for 3 weeks. Scans were acquired at baseline, and depressed patients were also scanned at the end of the treatment period.	Go/NoGo paradigm	Baseline scanes were assessed and remitters to treatment were distinguished from non-remitters by greater pre-treatment right dlPFC activation in the Go/NoGo contrast. Activation in cognitive tasks differentially predicted remission between medication type/class and was observed only during response inhibition.
25	Sladky et al. (2015)	**Aim:** To investigate the behavior of the serotonergic system during emotion processing and characterize the individual effects of the two citalopram enantiomers (*R* and *S*). **Hypotheses:** SSRI administration would increase connectivity between the orbitofrontal cortex and the amygdala leading to downregulation of amygdala activity.	Fifteen HVs. (5 female, mean age = 26.2). Randomized, placebo-controlled, double-blind design. Scans were acquired following a 10-day period of antidepressant medication (citalopram, escitalopram, or placebo).	Emotional face discrimination	(*S*)-citalopram, and likely other SSRIs, had mediating effects on effective connectivity within the prefrontal-amygdala network. Task-dependent inhibition of the amygdala was strongest during SSRI intake, with escitalopram showing slightly higher efficacy compared to citalopram.
26	Godlewska et al. (2016)	**Aim:** To assess whether neural markers of negative emotional processing changes predict later clinical response in depression. **Hypotheses:** A greater reduction in the neural response to fearful vs. happy facial expressions after 1 week of antidepressant treatment would be associated with increased response to treatment at week 6.	Thirty five participants with depression (20 female, mean age = 30.05) and 31 HVs. (17 female, mean age = 30). Depressed patients received 10 mg escitalopram for 6 weeks Scans were acquired at baseline, and patients were also scanned at weeks 1 and 6 post-treatment.	Emotional faces paradigm	Changes in neural processing of emotional information in the first week of escitalopram treatment in depressed patients predicted short-term (6 weeks) therapeutic response. Analysis of the fMRI data after 1 week of treatment alone was sufficient to predict outcome at 6 weeks with the same network of regions involved (insula, thalamus, amygdala, and cingulate).
27	Vai et al. (2016)	**Aim:** To investigate whether cortico-limbic connectivity associated with emotional bias measured before SSRI administration predicts the efficacy of antidepressant treatment in depressed patients. **Hypotheses:** Increased bottom-up connectivity in the amygdala and PFC would contribute to better antidepressant response to treatment, and this effect might underlie higher pre-treatment recruitment of cortical control on subcortical activity in responders.	Thirty depressed patients (19 female, mean age = 30.3) and 31 HVs. (18 female, mean age = 30.3). Patients received 10 mg escitalopram for 5–6 week. Scans were acquired at baseline and after treatment.	Gender discrimination paradigm	Pre-treatment, non-remitters, remitters, and HVs. had different functional characteristics. Following treatment, remitted depressed patients and HVs. had no differences. Non-responders to antidepressants may have different biological characteristics than responders. Baseline functional characteristics of the amygdala, ACC, and VLPFC predicted antidepressant response.
28	Wang et al. (2016)^χ^	**Aim:** To use fALFF to examine the effect of treatment with escitalopram on whole-brain spontaneous activity in depressed patients. **Hypotheses:** Escitalopram would modulate brain regions involved in emotional processing and regulation.	Thirty six first-episode, drug-naïve, non-comorbid depressed patients (11 female, mean age = 34.6) and 32 HVs. (11 female, mean age = 33.3). Depressed patients received 8 weeks of escitalopram 20 mg (*n =* 15), 15 mg (*n =* 4), or 10 mg (*n =* 1). Scans were acquired at baseline, and depressed patients were scanned at 8 weeks post treatment.	rsfMRI	Treatment-related changes in fALFF were found in several emotion-related brain regions (two regions in the PFC and in left putamen). fALFF values in these brain regions were significantly increased in depressed patients compared to HVs. at baseline and were reduced following treatment.
29	Outhred et al. (2016)^ϕ^	**Aim:** To explore the moderating effect of 5-HTTLPR on the impact of escitalopram during emotion regulation of negative emotional stimuli. **Hypotheses:** A linear (dose-response) relationship between 5-HTTLPR and left amygdala-right IFG FC during the reappraisal of negative images following a single dose of escitalopram relative to placebo was predicted. S/S allele carriers would display more positive coupling of these regions that L/L homozygotes during reappraisal of negative stimuli under escitalopram.	Thirty six right-handed female HVs. (mean age = 25). Randomized, double-blind, placebo-controlled study where participants received 20 mg oral escitalopram or placebo. Scans were acquired 4 h after drug administration.	Emotional processing task	With escitalopram, an increasing number of L alleles was associated with increasing negative left amygdala to right IFG FC during reappraisal. These preliminary findings extend the understanding of the pharmacogenetics of acute SSRI treatment and, if replicated, may provide important leads toward personalized medicine in affective disorders.
30	Cheng et al. (2017)	**Aim:** To investigate treatment effects in depression as measured using fMRI ALFF. **Hypotheses**: No specific hypotheses stated. An exploratory approach was followed to identify brain activation that could predict response to antidepressant treatment.	Seventy four depressed patients (22 male, mean age = 29.4) and 74 HVs. (22 male, mean age = 30.97). Depressed patients received escitalopram for 8 weeks, while HVs. received placebo. Patient scans were acquired at baseline, 5h post-administration (acute), and at weeks 4 and 8. HVs. received a single dose of placebo and were scanned at baseline, 5 h post-administration, and at week 8 post-treatment.	phfMRI	Abnormal rsfMRI ALFF was observed in depressed patients at baseline. Treatment with escitalopram induced connectivity changes both acutely (5h) and subchronically (8 wks). These changes were more profound in remitters than in non-remitters.
31	Takamura et al. (2017)	**Aim:** To investigate whether the ability of depressed patients to represent variable-sized monetary rewards in the striatum is disrupted. **Hypotheses:** Patients with depression would exhibit a disruption of the adaptive, modulatory response to variable reward amount. This abnormality would be related to SSRI treatment response.	Twelve depressed patients (6 female, mean age = 38.3) and 12 HVs. (6 female, mean age = 44.0). Depressed patients received escitalopram for 6 weeks. Scans were acquired within the first two weeks of treatment and at 6 weeks post-treatment.	Monetary Incentive Delay task	Depressed patients experienced motivational anhedonia at the neural level, showing reduced activation in brain areas including the ventral striatum and the putamen bilaterally. These decreased activations normalized with escitalopram treatment.
32	Spies et al. (2017)	**Aim:** To assess whether neural activity during an emotion discrimination task predicts early antidepressant effects and how these predictive measures relate to more sustained response. **Hypotheses:** No specific hypotheses stated. An exploratory approach was followed.	Twenty three depressed patients (16 female) received escitalopram for 4 weeks. Scans were acquired at baseline and at 4 weeks post-treatment.	Emotion discrimination paradigm	Deactivation of a cluster in the precuneus and PCC predicted depressive symptom reduction after 2 weeks of treatment. PCC and precuneus deactivation did not correlate with symptom improvement after 4 weeks of treatment. Early symptom improvement can be harnessed to optimize treatment regimens and patient care.
33	Wolf et al. (2018)	**Aim:** To use phfMRI to examine whether 5-HT influences activation in the emotion regulation networks during virtual violence by challenging the serotonergic system. **Hypotheses:** If 5-HT is involved in emotional regulation, SSRI administration would affect BOLD response (decreases in amygdala and mPFC) and also reduce activation in emotion regulation networks.	Thirty eight male HVs. (mean age = 24.7). Randomized, placebo-controlled, crossover design where placebo or citalopram were administered. Scans were acquired 3 h after drug administration.	Carmageddon paradigm	SSRI reduced responses to violent actions in right-hemispheric IFG and two mPFC clusters. The results suggest a link between functional response to virtual violent actions and serotonergic neurotransmission in emotion regulation networks.
34	Godlewska et al. (2018)	**Aim:** To explore the potential of pretreatment pgACC activity as a putative biomarker of treatment response. **Hypotheses:** Increased pgACC activation to masked sad facial expressions at baseline would predict later treatment response.	Thirty nine depressed patients (18 female, mean age = 28.5). Patients received 6 weeks of escitalopram treatment. Scans were acquired at baseline and at the end of the treatment period.	Backward matching task	Pretreatment neural activation of the pgACC in response to subliminal emotional information predicted short-term (6 weeks) therapeutic response to escitalopram in patients with depression.
35	Goldstein-Piekarski et al. (2018)^ψ^	**Aim:** To test the role of the DMN as a general and differential biomarker for predicting treatment outcomes in a large, unmedicated adult sample with depression. **Hypotheses:** Pretreatment connectivity with the PCC node of the DMN would predict general remission across antidepressants and differentially predict remission by type of antidepressant.	Eighty participants with depression (40 female, mean age = 32.8) and 34 matched HVs. (16 female, mean age = 31.5). Patients were randomized to three treatment groups: escitalopram (SSRI), sertraline (SSRI), or venlafaxine (SNRI) and received treatment for 3 weeks. Scans were acquired at baseline, and depressed patients were also scanned at the end of the treatment period.	rsfMRI	FC between the posterior and anterior regions of the DMN predicted remission following antidepressant treatment. Disrupted FC predicted poorer outcomes. At pretreatment, the profile of posterior to anterior DMN connectivity in eventual remitters closely resembled that of HVs. Pretreatment FC profiles hold promise for developing a neuroscience-informed approach to neuropsychiatric disorders and their management.
36	Meyer et al. (2019)	**Aim:** To determine neural prognostic predictors of antidepressant response and to gain insights into the temporal dynamics of antidepressant response by assessing neural mediators over the course of treatment. **Hypotheses:** No specific hypothesis stated. An exploratory approach was followed.	Twenty depressed patients (11 female, mean age = 31.5) and 66 HVs. (33 female, mean age = 26.3). Patients received escitalopram. Scans were acquired at baseline, 4–8 h, 4 weeks, and 8 weeks post-escitalopram treatment.	N-Back task	Changes to amPFC de-activation were crucial to mitigating depressive symptoms and specifically persistent residual cognitive impairments after clinical response. These results encourage the clinical use of fMRI for individual risk prediction of sub-optimal illness course.
37	Klöbl et al. (2020)	**Aim:** To find potential markers of treatment response. **Hypotheses:** No specific hypothesis stated. An exploratory approach was followed.	Thirty five depressed patients (16 female, median age = 26.96). Participants received either 8 mg of citalopram diluted in 8 ml saline or a comparable amount of saline as placebo IV over 8 min in a randomized, double-blind, crossover design. Scans were acquired during two MRI sessions 7 days apart.	rsfMRI	The short-term influence of citalopram on FC had predictive potential for certain clinician-rated, but not self-rated, instruments of antidepressant SSRI treatment response.
38	McMillan et al. (2020)	**Aim:** To investigate the role of the sgACC in the antidepressant effects of ketamine, and to use EEG-informed phfMRI to investigate the temporal dynamics of ketamine's neural effects. **Hypotheses:** Deactivation of the sgACC would mediate antidepressant response to ketamine.	Thirty depressed participants (13 female, mean age = 30.2). Placebo-controlled, crossover trial of ketamine (0.25 mg/kg bolus, 0.25 mg.kg infusion, 45 min) and remifentanil (1.7 ng/ml infusion). Simultaneous EEG and phfMRI were acquired prior and during drug/placebo administration.	phfMRI	No significant effects related to ketamine's antidepressant actions were detected with phfMRI. Decreased sgACC signal could most likely be attributed to noise.
39	Martens et al. (2021)	**Aim:** To identify differences in patterns of pre-treatment rsFC that may underlie response to antidepressant treatment. **Hypotheses:** No specific hypothesis stated. An exploratory approach was followed.	Thirty eight depressed patients (19 female, mean age = 30.2) and 32 HVs. (18 female, mean age = 30.3). Depressed patients received 10 mg escitalopram for 6 weeks. Scans were acquired at baseline and at 6 weeks post-treatment.	rsfMRI	Treatment responsivity was associated with enhanced rsFC of the right FPN with the posterior DMN, SMN, and somatosensory association cortex. Network connectivity is important in treatment response.

Greek letters (μ, ξ, π, ρ, ϕ, χ, ψ) indicate that the same samples were used across papers.

ACC, anterior cingulate cortex; ALFF, amplitude of low frequency fluctuations; amPFC, anterior medial prefrontal cortex; BOLD, blood oxygen level dependent; CBT, cognitive behavioral therapy; CCN, cognitive control network; dACC, dorsal anterior cingulate cortex; dlPFC, dorsolateral prefrontal cortex; DMN, default mode network; EEG, electroencephalography; fALFF, functional amplitude of low frequency fluctuations; FC, functional connectivity; fMRI, functional magnetic resonance imaging; FPN, frontoparietal network; HV, healthy volunteer; IED, intermittent explosive disorder; IFG, inferior frontal gyrus; mPFC, medial prefrontal cortex; OFC, orbitofrontal cortex; PCC, posterior cingulate cortex; PFC, prefrontal cortex; pgACC, pregenual ACC; phfMRI, pharmacological functional magnetic resonance imaging; rsFC, resting-state functional connectivity; rsfMRI, resting-state functional magnetic resonance imaging; SAD, social anxiety disorder; sgACC, subgenual anterior cingulate cortex; SMN, sensorimotor network; SNRI, serotonin-norepinephrine reuptake inhibitor; SSRI, selective serotonin reuptake inhibitor; VLPFC, ventrolateral prefrontal cortex.

## 3. Results

**Figure 2:**
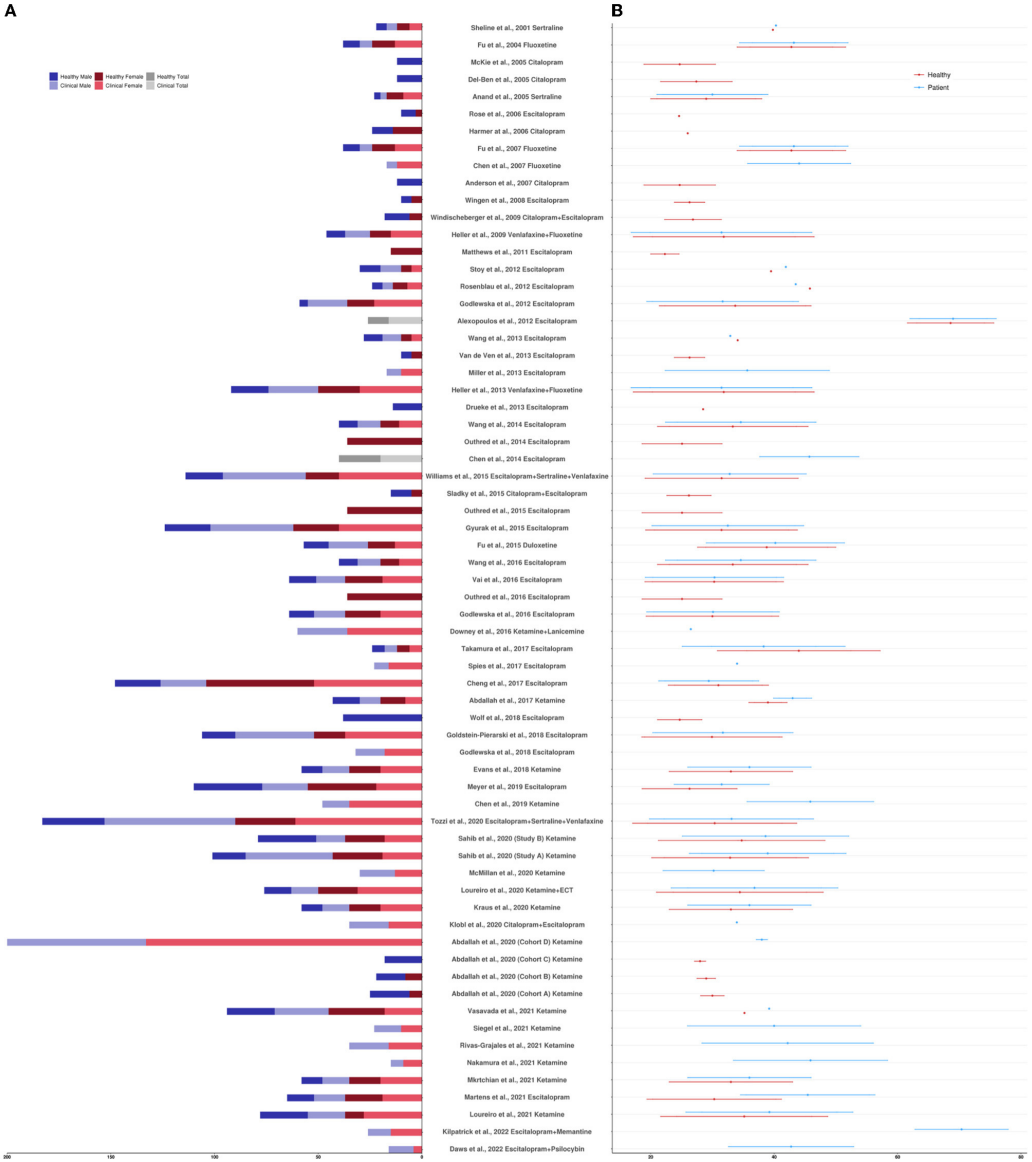
**(A)** Sample size reported per study by clinical designation and biological sex. **(B)** Sample age mean and standard deviation reported per study by clinical designation. If sample age was divided by treatment response or treatment arm, values were averaged; if sample age was not reported but an age recruitment range was provided, values were averaged. If multiple studies were reported in one paper, each study was counted separately.

The clinicaltrials.gov database identified 378 studies, 14 of which fulfilled our inclusion criteria. The ISRCTN Registry search identified 60 studies, 11 of which fulfilled our inclusion criteria. The 25 studies from the two clinical trial databases are summarized in Table 1. These studies included patients with depression, including bipolar depression and treatment-resistant depression (TRD), as well as healthy volunteers, depending on their aims and study design. When compounds with antidepressant action were entered as search terms, 39 additional experimental research studies that were not registered as clinical trials were identified that were relevant to this review (see Table 2). The ratio of clinical trials to research studies, as well as the imaging techniques used in each study, are presented in Figures 1A, B. The sample size and biological sex balance of all included studies are presented in Figure 2.

Predictably, most of the included studies investigated SSRIs, which are the most commonly prescribed treatments for depression (Figure 1C). Compounds with a glutamatergic mechanism of action, mainly ketamine, were the second most frequently identified class of medication with antidepressant action. The SSRI studies mainly focused on the mechanism of action of these compounds as treatments for depression and often reported outcomes that involved the amygdala. The amygdala was a common target area for all investigational compounds for depression, with 67% of the clinical trials and research studies included in this review reporting outcomes in this region (Figure 1C).

### 3.1. SSRIs

#### 3.1.1. The amygdala

Twenty-two of the 48 studies that investigated antidepressants with serotonergic action and reported findings in the amygdala used task-based fMRI involving discrimination of emotional faces. These studies found increased amygdala activation at baseline in response to negative stimuli in individuals with depression vs. healthy volunteers [as an example, see (Godlewska et al., 2012). Administration of SSRIs, mainly sertraline and escitalopram, decreased amygdala activation in response to fearful faces (Sheline et al., 2001; Godlewska et al., 2012, 2016), sad faces (Fu et al., 2004), and negative pictures (Rosenblau et al., 2012), and this decrease positively correlated with reduced depressive symptoms six to eight weeks post-treatment. This trend toward normalization of amygdala activation in response to SSRIs was observed as early as seven days after treatment, when no change in depressive symptoms had yet been observed (Godlewska et al., 2012). Baseline amygdala activation (Williams et al., 2015), as well as amygdala connectivity with the anterior cingulate cortex (ACC) and ventrolateral prefrontal cortex (VLPFC) (Godlewska et al., 2018) also predicted treatment response to specific SSRIs.

In contrast to studies of individuals with depression—which, with few exceptions, have targeted the amygdala—studies of healthy volunteers are much more exploratory in their approach. One pharmacological fMRI (phfMRI) study in healthy volunteers found that activity in the caudate, subgenual and anterior cingulate, hippocampus, amygdala, and frontal cortex increased after a single citalopram infusion compared to placebo (McKie et al., 2005). Other studies that assessed amygdala activity at peak blood concentration levels for various SSRIs found that reduced activation was observed in response to negative stimuli in healthy volunteers (Del-Ben et al., 2005; Anderson et al., 2007; Outhred et al., 2015, 2016). Finally, when healthy volunteers received compounds with a serotonergic mechanism of action for 7-10 days, reduced amygdala and parahippocampal activation was observed in response to emotional stimuli (Harmer et al., 2006; Windischberger et al., 2010).

#### 3.1.2. The anterior cingulate cortex (ACC) and striatal areas

Four studies examined the effects of SSRIs on the activation and connectivity of the ACC in depression. These studies found that baseline activity and connectivity in the ACC in response to emotional and negative stimuli successfully predicted response to treatments for depression, highlighting the potential importance of studying the ACC as a brain area that could predict treatment response (Chen et al., 2007; Miller et al., 2013; Godlewska et al., 2016, 2018).

When activations in the striatum were examined, unmedicated patients with depression demonstrated hypoactivity in the VS during the anticipation of positive and negative monetary rewards (Stoy et al., 2012). This hypoactivation was reversed after 6 weeks of treatment with escitalopram.

#### 3.1.3. The default mode network (DMN) and connectivity between other brain areas

Individuals with depression showed increased DMN connectivity that reduced in response to antidepressant treatment, a decrease that correlated with symptom improvement (Alexopoulos et al., 2012; Goldstein-Piekarski et al., 2018; Kilpatrick et al., 2022). Psilocybin, an agent with serotonergic action, also produced disorganization of the DMN along with other brain networks, a finding that correlated with improved depression outcomes (Daws et al., 2022). Finally, both acute and longer-term (three weeks) treatment with escitalopram reduced DMN connectivity in healthy volunteers, a finding that is in line with the effects that the drug produces in depression (Matthews et al., 2010; van de Ven et al., 2013).

Concerning connectivity between other brain areas and networks, SSRIs increased corticolimbic connectivity in patients with depression, 6 weeks post-treatment (Anand et al., 2005), while frontostriatal connectivity changes induced by antidepressant treatment correlated with positive day-to-day emotion during an emotional processing task (Heller et al., 2013a).

### 3.2. Antidepressants with glutamatergic action

Studies investigating the role of glutamatergic agents have mainly focused on the effects of the N-methyl-D-aspartate (NMDA) glutamatergic modulator ketamine as an antidepressant treatment.

*The amygdala* Loureiro et al. (2020) showed that both ketamine and electroconvulsive therapy decreased amygdala reactivity to positive and negative emotional face processing. Additionally, in an emotional judgement task analysis, the researchers found that, at 24 h post-administration, ketamine decreased activation of the amygdala along with the insula, ACC, and ventral tegmental areas (Sterpenich et al., 2019).

#### 3.2.1. The anterior cingulate cortex and striatal areas

Compared to baseline, ketamine enhanced neural response to positive emotion in the right caudate of individuals with depression undergoing an emotional processing task (Murrough et al., 2015). Ketamine also decreased activation in the ACC and ventral tegmental areas during an emotional judgement task (Sterpenich et al., 2019). Finally, in a study by Downey et al., it was shown that ketamine and lanicemine, both increased activation of the sgACC and rostral ACC.

#### 3.2.2. The default mode network (DMN) and connectivity between other brain areas

A single ketamine infusion normalized global brain connectivity in individuals with depression (Abdallah et al., 2017), though this finding was not replicated by Kraus and colleagues (Kraus et al., 2020). Normalization of the connectivity between the insula and the DMN (Evans et al., 2018), the subgenual ACC (sgACC) and the DMN (Nakamura et al., 2021; Siegel et al., 2021), the habenula and occipital and temporal areas (Rivas-Grajales et al., 2021), as well as fronto-striatal connectivity (Mkrtchian et al., 2021) was also observed 24 and 48 h after a single ketamine infusion. When the effects of 4 weeks of ketamine treatment on brain connectivity were examined, decreased functional connectivity between the cerebellum and the salience network was observed in individuals with depression (Vasavada et al., 2021); increased functional connectivity between the cerebellum and the striatum prior to treatment predicted remission (Sahib et al., 2020a). 4 weeks of ketamine treatment (Vasavada et al., 2021) also significantly increased functional connectivity between limbic regions and the central executive network while decreasing connectivity between the left amygdala and the salience network (Siegel et al., 2021).

### 3.3. Other compounds with antidepressant action

In one study of the serotonin-norepinephrine reuptake inhibitor (SNRI) duloxetine, unmedicated individuals with depression who received 12 weeks of duloxetine showed normalized activation in the amygdala and ACC, a finding that was linked to symptom reduction (Fu et al., 2015). Studies of venlafaxine, another SNRI, found that venlafaxine had no effect on amygdala activation after 2 months (Heller et al., 2013a) and 6 months of treatment (Heller et al., 2013b), and that amygdala activity at baseline did not correlate with symptom changes and negative affect regulation (Heller et al., 2009).

## 4. Discussion

This review sought to examine the use of fMRI as an outcome measure in clinical trials and research studies that focus on depression. Most of the studies seeking to understand the changes in brain activity and connectivity that accompany successful antidepressant treatment and predict treatment response have, to date, focused on SSRIs and, to a lesser extent, ketamine. Our discussion will focus on the amygdala and on ketamine as two different avenues for assessing what the field has learned from the use of fMRI in clinical trials and research studies in depression.

Almost half of the SSRI studies exploring the effects of serotonergic compounds in depression (20 out of 44 in our review) focused on the amygdala, a brain area involved in the processing of emotionally valenced stimuli. Research in individuals with depression has consistently observed increased activation in the amygdala during the presentation of fearful and negative stimuli (Drevets et al., 2008). Furthermore, successful treatment for depression has tended to normalize this amygdala hyperactivity, often prior to any detectable symptom changes (Godlewska et al., 2012, 2016; Rosenblau et al., 2012; Vai et al., 2016). Decreased amygdala activity was primarily observed after SSRI administration, although it remains unclear whether this effect is specific to SSRIs or whether it also extends to antidepressant treatments with different mechanisms of action (Williams et al., 2015). In addition, the effect of antidepressant treatment on amygdala activation appears to be specific to the type of stimulus that triggers the activation of that brain area. For instance, amygdala activation increased at baseline in response to fearful/sad faces (Godlewska et al., 2016), a finding in line with depression being characterized by increased emotional response to negative affect (Godlewska et al., 2016) and associated with heightened amygdala response.

The fact that chronic and acute SSRI administration produce similar effects on the amygdala of healthy volunteers and individuals with depression (Harmer et al., 2006; Windischberger et al., 2010; Outhred et al., 2015) offers insight into the antidepressant mechanism of action of those compounds. It also underscores both that the amygdala is a key area for antidepressant response and that fMRI is a useful tool for understanding antidepressant drug action. Interestingly, the anxiolytic drug lorazepam also appears to decrease amygdala activation (Paulus et al., 2005), further highlighting the importance of this area as a potential “hub” for drug action in mental health.

Although the studies summarized here consistently observed decreased amygdala activation following treatment with SSRIs, they did not determine whether antidepressant response was specific to the right or left amygdala or appeared bilaterally. If the amygdala is to be used as an fMRI biomarker to determine treatment response, more precise localization of these effects is required. In addition, although most of the amygdala studies to date have focused on emotional processing, they use different fMRI tasks to examine response. While observation of amygdala activation under different experimental conditions strengthens the idea that the amygdala plays a critical role in antidepressant drug action and highlights fMRI as a powerful tool for drug research, consensus on a single paradigm that could be used to determine drug effects in the amygdala could be useful for studies seeking to establish its validity as a marker of drug efficacy. Finally, it is worth highlighting that although the majority of the task-based fMRI studies for SSRIs have focused on emotional processing tasks, there are other tasks including reward processing (for example, Stoy et al., 2012), response inhibition (see Gyurak et al., 2016) and working memory (for review on the n-back task see Nikolin et al., 2021) that have also been used in addition to emotional processing or in isolation in order to examine different brain processes that are affected in depression. These tasks could help in the identification of novel drug targets as well as contribute to the better understanding of the depression-related brain changes. Finally, when incorporated in to clinical trials, they provide a more holistic characterization of the mechanism of action of different antidepressant treatments.

The NMDA receptor antagonist ketamine is the most widely investigated of the glutamatergic antidepressants. Ketamine is a commonly used anesthetic and, at sub-anesthetic doses, a potent and rapid-acting treatment for depression (Berman et al., 2000). It should be noted, however, that although ketamine's antidepressant efficacy is a rather well-replicated finding (McIntyre et al., 2020)—especially in cases of TRD—research around the neuronal pathways that underlie ketamine's actions and the relevant brain areas involved in antidepressant response to ketamine remain unknown. Indeed, ketamine's antidepressant effects on brain activity and connectivity are complex and difficult to decipher compared to serotonergic agents. fMRI studies that investigated ketamine as an antidepressant focused on drug-induced changes in brain activity and connectivity; in particular, these studies found that ketamine normalized the connectivity changes associated with depression (Abdallah et al., 2017; Evans et al., 2018; Mkrtchian et al., 2021). Each study, however, focused on different networks and/or brain areas implicated in the symptoms of depression and, as a result, few findings have yet been replicated across studies. In addition, most of these studies focused on the 24 h post-ketamine administration timepoint, when the drug's antidepressant effects peak; nevertheless, changes in brain activity and connectivity that precede these effects might also be important markers for ketamine's antidepressant actions. Another key point is that, although some studies of multiple ketamine infusions have been conducted (Sahib et al., 2020a; Vasavada et al., 2021), most findings are drawn from analyses conducted on samples collected after a single infusion. Further research and replication in other cohorts is needed to confirm the ketamine fMRI findings.

Task-based fMRI studies that aim to characterize ketamine's antidepressant mechanism of action have mainly focused on emotional processing and reward tasks and identified decreased amygdala activity after ketamine administration (Siegel et al., 2021). This finding is in line with the antidepressant effects observed for SSRIs and SNRIs, further underscoring the potential role of the amygdala as a biomarker for determining antidepressant drug efficacy with fMRI. Increased striatal activity has also been observed with task-based fMRI post-ketamine administration (Sterpenich et al., 2019). Although few such studies exist, their results are in line with literature reporting decreased striatal response in individuals with depression associated with blunted responses to reward and feedback; the findings are also in line with ketamine's positive effects on anhedonia (Wilkowska et al., 2021).

Despite these intriguing early findings, many questions need to be addressed before fMRI can successfully be used to identify brain areas that could determine successful antidepressant response to ketamine. For instance, are the changes in brain activity and connectivity that occur during acute ketamine administration relevant and necessary for determining antidepressant response to ketamine? Furthermore, given that ketamine is mainly used to treat TRD, a better understanding of the brain changes that characterize TRD is necessary. For example, research suggests that individuals with TRD may have a different brain response to ketamine than those with other forms of depression (Sun et al., 2022), suggesting that different brain areas that might serve as biomarkers to determine treatment response in TRD vs. other forms of depression (Carboni et al., 2021).

Apart from the biophysical considerations, fMRI data quality, acquisition parameter selection, trial type and size, may also affect the detection and reliability of the markers identified. Although a detailed review of these choices is outside the scope of the current discussion (see Bodurka et al., 2007; Noble et al., 2017; Raimondo et al., 2021), it is worth pointing out that fMRI data quality can be well-monitored prospectively, during a study, if appropriate quality control measures are in place (for example Reynolds et al., 2022). Technical developments in fMRI have increased the potential to localize small brain structures. The fundamental requirement of adequate temporal signal-to-noise ratio (tSNR) to detect them is dictated by several variables including field strength, spatial and temporal resolution, and scan length (Murphy et al., 2007) and consensus suggestions for the optimal values of these parameters are still not in place. For resting state scans, optimal scan lengths have been studied and it was shown that 9–12 min of scanning is sufficient to reach a plateau for inter-session reliability (Birn et al., 2013) while five, repeated 5 min sessions of resting state scanning, pooled together, is enough to provide “fair” reliability (Noble et al., 2017) with no major site, scanner manufacturer, or day-of-scan effects found. For task based scans, there has been a recent suggestion that increasing the number of event trial sample size has nearly the same impact as subject sample size on statistical efficiency (Chen et al., 2022). From the above, it is safe to conclude that unsurprisingly the underlying data quality and quantity would have a significant impact on the ability to detect and identify markers. In that respect, current trends toward openly sharing raw data could enable researchers to have more confidence in the origin of their results.

Taken together, the fMRI findings reviewed above suggest that successful antidepressant response is reflected in a normalization of the brain activity and connectivity deficits associated with depression. As a result, areas that present with altered brain fMRI activation in depression could be used as potential treatment targets in clinical trials. Such targeted use of fMRI would require that the changes in brain activity and connectivity that underlie depression and depressive symptoms be consistently observed and well-characterized in individuals with depression.

## 5. Future directions

fMRI research that aims to understand changes in brain activity and connectivity in individuals with depression as well as healthy volunteers is often obscured by our relatively poor understanding of the long-term effects of antidepressant medication on brain activity and connectivity. A meta-analysis of resting-state studies in depression that took antidepressant treatment into account (Javaheripour et al., 2021) suggested that the depressed brain may not be as well-characterized as previously believed. The effects of natural aging on brain activation and connectivity should also be taken into account, especially in studies where there is a broad age range between participants (Kaufmann et al., 2019).

Because of its rapid antidepressant effects, ketamine may be a particularly useful tool for investigating antidepressant action on brain circuits, thus bypassing some of the confounding factors associated with functional imaging research. Its quick action enables researchers to measure a baseline state with significant symptomatology, the acute drug effects associated with ketamine, and the subsequent symptom reduction that occurs within hours. The ability to acquire successive images of an individual's changing state on such a short timescale increases the likelihood that the measured changes are due to mood improvement from the drug rather than other environmental (e.g., hydration) or longitudinal changes (e.g., lifestyle changes, aging) that can confound imaging findings. Studying the antidepressant mechanisms of ketamine could also improve our understanding of antidepressant drug action in general, given that the pathways and brain areas that mediate antidepressant effects—for example the amygdala (Sheline et al., 2001; Godlewska et al., 2012, 2016; Loureiro et al., 2020) and DMN (Matthews et al., 2010; van de Ven et al., 2013; Nakamura et al., 2021; Siegel et al., 2021)—appear to be shared between different antidepressants. Within that framework of shared pathways between drugs and mood disorders, employing an approach such as the one proposed by the Research Domain Criteria Initiative (RDoC) could be particularly useful. The RDoC framework provides an organizational structure for research that is based on key domains of neurobehavioral functioning that are shared across several mental health disorders (Cuthbert and Insel, 2013). Research planned with these criteria in mind, would lead to a common framework for results reporting which could increase the reproducibility of findings.

Concerning the fMRI modalities, task-based or resting-state, that different studies use to examine the effects of antidepressant treatments, resting-state fMRI, seems to be producing consistent findings, especially in relation to the brain connectivity responses induced by ketamine. However, it should be noted that imaging studies in general and particularly those involving resting-state fMRI, present the challenge of disentangling the antidepressant effects of pharmacological treatments from the neurovascular ones (De Simoni et al., 2013). This could be mitigated by measuring peripheral physiological changes and/or using multi-echo sequences or concurrent EEG monitoring (McMillan et al., 2020). Additionally, there are many different approaches that are currently used to analyze resting-state fMRI data, including looking at brain networks and/or individual regions of interest (ROIs). Different pharmacological studies, employ one or more of these approaches, which would best fit with the study design and hypotheses, however, this limits the replicability of the resting-state fMRI findings. A battery of standardized analyses that could be included to all pharmacology studies that aim to exam drug efficacy, applied along with exploratory approaches, could provide with findings that are replicable and help better characterize and understand drug effects.

Task-based fMRI studies use different paradigms to target specific brain areas and to examine drug effects in those regions. However, a large variety of tasks—as well as many variations of the same task—are used to elicit specific responses from participants. This is an excellent way to explore the neural responses that underlie different tasks and different drugs, but a high number of task variations also results in a lack of replicability. A recent review and meta-analysis found that commonly used fMRI tasks do not currently have the test-retest reliability necessary for biomarker discovery or brain-behavior mapping (Elliott et al., 2020). In this context, the field would benefit from developing robust task paradigms that could be implemented in the scanner and parallel the effectiveness of clinically developed rating scales. In addition, traditional tasks have been designed to reduce inter-subject variability for stability in group averages and may thus not reflect changes within an individual with repeated scans. In order to best capture individual dynamics, the use of more ecologically valid tasks has been proposed, including using naturalistic stimuli (Finn et al., 2020).

Another area that could be re-examined in order to enhance the reliability of fMRI measures as well as the quality of fMRI data—and especially task-based fMRI measures—would be a trade-off between the number of participants and the amount of data collected per participant that is needed to make a particular inference (Naselaris et al., 2021). Because of their specialized populations and the additional burden and time required to complete a full trial, clinical studies take a long time to accrue enough participants for imaging trials. As a result, collecting more data from a smaller number of participants might be a promising way to improve the reliability of fMRI (Gordon et al., 2017; Gratton et al., 2018; Elliott et al., 2019), employ better task controls, collect data at many different time points as well as increase the length of data that are collected. Forming complete models from a relatively small number of individuals with dense, high quality fMRI sampling (akin to N-of-1 trials) may provide clinically useful imaging information and generalizable results that may help with research reliability and biomarker development. A smaller study sample could also allow for the better characterization of the study population in terms of symptoms. In clinical populations, comorbidities are a factor that is almost impossible to avoid. Depression for example, is often accompanied by anxiety or trauma-related disorders (Kupfer and Frank, 2003). Although, a mixed sample would add to the ecological validity of the study, it could impact the replicability of the study findings as well as make it difficult to link the brain changes that are observed after the drug administration to a specific disorder. The type of dense sampling that we proposed above, also lends itself favorably to deep phenotyping of individual responses when multiple modalities are acquired—for instance, from genetics, blood, behavioral data, and a variety of imaging modalities [e.g., magnetoencephalography (MEG), MRI, positron emission tomography (PET)] which could lead to more direct links between disorders and drug effects as well as enable the recruitment of more homogenous clinical populations. However, this dense sampling method may be difficult for clinical populations, where the burden that these interventions pose could add to participants' health and mood challenges; implementation of any such methods should thus be carefully considered. Alternatively, or in addition to this approach, large multi-site studies that could assemble large data sets across diverse population (Spellman and Liston, 2020) could also help increase the power and reliability of imaging studies, although they face the challenge of coordinating data collection from multiple sites while ensuring data quality and consistency.

Finally, data sharing between different labs and research centers would also enable the analyses of larger data sets that could increase the reliability of findings as well as help overcome the common problem of small sample sizes that most clinical trials and pharmacology research studies are faced with.

However, several positive steps have already been made in this direction, with studies beginning to employ a standardized and uniform way of organizing imaging data (Gorgolewski et al., 2016; Niso et al., 2018). Such efforts ensure that data are readily available for sharing between different acquisition centers, but also between researchers, making their analysis much easier and more robust and thus more reliable.

## 6. Conclusions

This review sought to examine the use of fMRI in clinical trials and research studies of compounds that target depression. Much of the work conducted to date has identified the amygdala as a particularly promising target region for antidepressant treatments. Indeed, most studies in this review found a consistent decrease in amygdala activity following antidepressant treatment. This review also highlighted the potential role of ketamine as a tool for studying antidepressant effects with fMRI, given that ketamine administration not only leads to very rapid antidepressant effects but consistently normalizes brain activity and connectivity in depression. These findings highlight the considerable promise of fMRI for studying and developing drugs targeted at mental health conditions in general and depression in particular. Nevertheless, a gap persists between the extensive use of fMRI in research and its systematic and primary use in clinical trials-related research. Efforts to develop more effective fMRI tasks and adapt existing study designs would help harness the advantages of fMRI and improve our ability to translate research findings into clinical trial outcomes.

## Author contributions

VK: conceptualization, research, analysis, and writing. JE: conceptualization, analysis, and writing. CP: research, analysis, and writing. CZ: analysis, editing for critical intellectual content, and supervision. All authors contributed to the article and approved the submitted version.
