# Supplementary material for: Review: The use of functional magnetic resonance imaging (fMRI) in clinical trials and experimental research studies for depression
Source: Front Neuroimaging. 2023 Jun 27;2:1110258. doi: 10.3389/fnimg.2023.1110258 (PMC10406217; doi:10.3389/fnimg.2023.1110258)
Supplement: Supplementary file 1 [file Data_Sheet_1.docx]

**Identification of studies via databases and registers**

Records identified from:

clinicaltrials.gov, ISRCTN, and PubMed databases (n=3)

**Identification**

Records screened

(n=378, clinicaltrials.gov)

(n=60, ISRCTN)

(n=39, PubMed)

Records excluded. No publications associated with registered clinical trials (n = 100).

Reports sought for retrieval

(n=308, clinicaltrials.gov)

(n=30, ISRCTN)

(n=39, PubMed)

Reports not retrieved

(n = 0)

**Screening**

Reports excluded:

Not relevant to depression (n=150)

Insufficient information on treatment (n = 143)

Insufficient information on sample selection criteria (n=20)

Reports assessed for eligibility

(n=308, clinicaltrials.gov)

(n=30, ISRCTN)

(n=39, PubMed)

Studies included in review

(n=14, clinicaltrials.gov)

(n=11, ISRCTN)

(n=39, PubMed

**Included**

*From:*  Page MJ, McKenzie JE, Bossuyt PM, Boutron I, Hoffmann TC, Mulrow CD, et al. The PRISMA 2020 statement: an updated guideline for reporting systematic reviews. BMJ 2021;372:n71. doi: 10.1136/bmj.n71

For more information, visit: <http://www.prisma-statement.org/>
